# The role of chemotherapy and operation on lymphocytes accumulation in peripheral blood obtained from patients with oral squamous cell carcinoma

**DOI:** 10.1186/s40064-015-1485-6

**Published:** 2015-11-12

**Authors:** Tao Yu, Ping Guo, Yingying Wu, Jiafeng Zhao, Lichun Wu, Chunhua Li, Kun Liu, Guiquan Zhu, Jin Chen, Chuan Xu, Yongcong Cai, Jifeng Liu, Zhaohui Wang

**Affiliations:** Department of Head and Neck Oncology Surgery, Sichuan Cancer Hospital, No.55, Sec.4, Renminnan Road, Chengdu, 610041 Sichuan People’s Republic Ofchina; State Key Laboratory of Oral Diseases, West China School of Stomatology, Sichuan University, Chengdu, 610041 Sichuan People’s Republic Ofchina; Department of Clinical Laboratory, Sichuan Cancer Hospital, No.55, Sec.4, Renminnan Road, Chengdu, 610041 Sichuan People’s Republic Ofchina

**Keywords:** Oral squamous cell carcinoma, Immune subset, Chemotherapy, Radical operation

## Abstract

The “Cancer Immunoediting” concept has provided critical insights suggesting dual functions of immune system during the cancer initiation and development. However, the dynamics and roles of CD4^+^ and CD8^+^ T cells, CD19^+^ B cells, and CD56^+^ NK cells in the patients with oral squamous cell carcinoma during treatment remain unclear. A total of 43 patients with OSCC were divided into different groups according to different clinical factors (TNM staging, pathological patterns, age and genders) for assessment of relations with CD3^+^CD4^+^ T cells, CD3^+^CD8^+^ T cells, CD3^−^CD19^+^ B cells and CD3^−^CD16^+^CD56^+^ NK cells and different chemotherapy and radical operation. The expression of CD3^+^CD4^+^ T cells were significantly increased in advanced tumor stage, large tumor size and positive lymph nodes metastasis, compared to that in early groups. The accumulation of CD3^+^CD4^+^ T cells were significantly increased in OSCC patients received 2 cycles CT and radical operation. Moreover, the accumulation of CD3^+^CD8^+^ T cells were significantly decreased in OSCC patients received 2 cycles CT and radical operation. The distribution of circulating CD3^−^CD19^+^ B cells was related with radical operation in patients with OSCC. This study indicate that CD4^+^ T cells have opposing roles in OSCC progression and outcomes, which provides new insights relevant for the development of effective cancer immunotherapeutic approaches. 2 cycles TP regime chemotherapy and radical therapy may contribute to increase the effects of anti-tumor immunity on patients with OSCC.

## Background

Increasing evidence suggests that interplay between immune cells and tumor cells exerts a major influence on oral tumor development and progression (Sahingur and Yeudall 2015). Furthermore, recent “Cancer Immunoediting” concept provided insights that immune system has both immune surveillance and tumor promotion effects during the cancer development (Dunn et al. 2004; Schreiber et al. 2011). In recent years, with the rapid development of molecular biology and immunology and the multidisciplinary cross integration, achievements have been made in the research of mutual regulation among immunological cells in tumor tissues (de Cos Escuin 2014; Liu et al. 2014). Of the examination indexes for diagnosing the development, progression and prognosis of tumors, the detection of lymphocyte subgroups in peripheral blood is the most popular one in clinic (Liu et al. 2014; Jozwik et al. 2015). It is established that an effective anti-tumor immune response requires the involvement of both CD4^+^ and CD8^+^ T cells (Dunn et al. 2004; Schreiber et al. 2011). The role of CD4^+^ T cells in anti-tumor immunity has recently been extensively studied in both pre-clinical animal models and clinical cancer patients. CD4^+^ T cells are critical for priming of tumor-specific CD8^+^ T cells and for the secondary expansion and memory of CD8^+^ T cells as well (Janssen et al. 2003). The immune status of patients is commonly evaluated in terms of circulating lymphocyte subsets, including CD3^+^ T cells, CD3^+^CD4^+^ T cells, CD3^+^CD8^+^ T cells, CD19^+^ B cells and CD56^+^ NK cells (Plonquet et al. 2007; Ersvaer et al. 2007). There are some works on the role of the T cells in head neck squamous cell carcinoma. Boucek et al. analyzed the blood samples from 112 patients with head and neck squamous cell carcinoma lymphocyte subpopulations (CD3^+^; CD3^−^CD16^+^CD56^+^; CD4^+^; CD8^+^; CD19^+^; CD4^+^CD45RA^+^). The results demonstrated that the percentage of CD8^+^ cells increased and the CD4/CD8 ratio decreased with tumor grade. The ratio of B lymphocytes decreased in patients with locoregional metastases (11.25 versus 9.22 %). Treg (15.2 %) and CD4^+^ cells (45.3 %) increased, while NK cells (11.8 %) decreased in HNSCC patients compared to controls (9.0, 38.1 and 15.8 %, respectively) (Boucek et al. 2010). Starska et al. performed the cytofluorymetric analysis of the early (CD69^+^, CD71^+^) and late activation markers (${\text{CD25}}_{\text{high}}^{ + }$, CD26^+^, HLA/DR^+^) expression on T CD3^+^CD4^+^ and CD3^+^CD8^+^ cells subpopulations in mixed cellular cultures of freshly isolated tumor cells (MLTMC) and non-cancerous normal epithelial cells (MLNCC) in 55 cases of squamous cell laryngeal carcinomal. Meanwhile, the whole peripheral blood concentrations of IL-10 and IFN-g in 21 and 72 h of experiments were also measured by ELISA. In addition, Starska et al. identified that the expressions of CD69^+^ and CD71^+^ antigens on T CD3^+^CD4^+^ and CD3^+^CD8^+^ cells as well as CD4^+^HLA/DR^+^ markers were higher for pT3 and pT4 tumors, in comparison with pT2 carcinomas. These studies were similar with our present study (Starska et al. 2011a, b). However, few studies were focused on the dynamic correlation between circulating lymphocyte subsets and different treatments in patients with oral squamous cell carcinoma (OSCC). Furthermore, the influence of chemotherapy (CT) and radical operation on circulating lymphocyte subsets of patients with OSCC remains unclear (Lau et al. 2007; Hsu et al. 2010). In the current study, the correlations between circulating immune subsets and pre- and post-treatment as well as the clinical characteristics were investigated in OSCC patients.

## Methods

### Patients

The study protocol was approved by the ethical committee of the Sichuan Cancer Hospital, China and was performed in accordance with the ethical standards laid down in the 1964 Declaration of Helsinki and its later amendments. The clinical data, including age, gender, location, size of tumor, nodal status, histologic type, and treatment were obtained in department of Head and Neck Oncology Surgery, Sichuan Cancer Hospital from January 2013 to December 2014. The diagnosis depended on the pathological finding and every case was restaged according to the UICC TNM Classification of Malignant Tumors (The seventh edition). All cases were histologically graded into well, intermediate and poor grade OSCC. The primary locations of tumor were from tongue, buccal, and the floor of mouth. The patients with T_1_/N_0_ OSCC only received radical operation (extensive resection). The patients with T_2–4_/N_1–3_ OSCC received 2 cycles TP regime chemotherapy and then radical operation, including extensive resection of the primary tumor, partial resection of maxillary/mandible, and functional neck dissection (Level I–V). The TP chemotherapy regime was comprised 30 mg/m^2^ cisplatin on days 1–3 plus 100 mg/m^2^ paclitaxel on days 1.

### Immune subset measurement

Before treatment and 7 days after 2 cycles CT or radical operation, analysis of total lymphocytes and subsets was performed on whole blood samples (100 μL; 5 × 10^5^–1 × 10^6^ cells) collected into EDTA-treated tubes. Blood was stained with the following conjugated murine antihuman monoclonal antibodies (Becton–Dickinson, San Jose, CA, USA): CD4 (T helper cells), CD8 (cytotoxic T cells), CD19 (B cells) and CD56 (natural killer cells). The mixtures were incubated for 30 min in the dark, washed with PBS (3 mL) and centrifuged at 1500 rpm for 5 min. Cells were analyzed using a multiparametric four-color flow cytometer (BD FACSCalibur, CA, USA). Percentages of circulating CD3^+^CD4^+^ T cells, CD3^+^CD8^+^ T cells, CD3^−^CD19^+^ B cells and CD3^−^CD16^+^CD56^+^ NK cells were quantified, and CD4^+^/CD8^+^ ratios were also calculated with Cellquest software (BD Biosciences).

### Statistical analysis

Data are expressed as mean ± standard deviation (SD). The significance of difference between groups was determined by paired or unpaired two-tailed Student’s *t* test or the one-way analysis of Variance (ANOVA). Difference was considered significant for p values less than 0.05.

## Results

### The clinical data

Patients’ age ranged from 23 to 77 years (median 58.8), with 21 cases belonging to the >60 age group, accounting for 48.8 % of all patients. The locations of primary tumor were tongue, buccal, and the floor of mouth, respectively. There were 34 male and 9 female subjects. The size of the primary tumor was 1.5–6.0 cm (median 4.5). According to the seventh edition of the TNM classification, 17 cases were clinically classified as T_1–2_ and 26 cases were clinically classified as T_3–4_. In addition, 19 cases were clinically classified as N_0_ and 24 cases were clinically classified as N_1–3_. Only two cases had lung and bone metastasis. The primary diagnosis was performed by incisional biopsy. 21 were pathologically classified as well differentiated OSCC, 14 cases as intermediate differentiated OSCC, and 8 cases as poor differentiated OSCC. Clinical data of all cases are summarized in Table 1.

**Table 1:** Correlations between the percentage of different lymphocyte subgroups and clinicopathologic characteristics in OSCC

Groups	N	CD3^+^CD4^+^	CD3^+^CD8^+^	CD3^−^CD19^+^	CD3^−^CD56^+^
Gender					
Male	34	37.27 ± 8.29	27.79 ± 5.57	10.24 ± 5.08	18.75 ± 9.00
Female	9	36.56 ± 7.88	26.44 ± 4.98	10.58 ± 3.76	16.49 ± 8.66
Age					
≤40	3	30.33 ± 4.04	22.33 ± 3.51	9.50 ± 2.17	24.60 ± 4.85
40–60	19	39.68 ± 6.95	29.58 ± 4.74^a^	11.04 ± 5.63	18.48 ± 9.42
≥60	21	35.76 ± 8.84	26.38 ± 5.55	9.77 ± 4.29	17.19 ± 8.73
Location					
Tongue	21	38.1 ± 8.09	27.33 ± 5.17	11.03 ± 5.89	19.14 ± 8.97
Floor of mouth	13	36.69 ± 8.04	27.92 ± 5.82	9.60 ± 3.35	17.06 ± 9.00
Buccal	9	35.44 ± 8.93	27.33 ± 6.02	9.66 ± 3.85	18.02 ± 9.31
Tumor size					
T_1–2_	17	29.06 ± 3.44^b^	22.65 ± 3.10^c^	1048 ± 4.52	15.82 ± 8.39
T_3–4_	26	42.39 ± 5.49	30.69 ± 4.08	10.20 ± 5.06	19.89 ± 8.98
Nodal status					
N_0_	19	30.58 ± 5.04^d^	24 ± 4.66^e^	1045 ± 4.78	17.48 ± 9.34
N_1–3_	24	42.29 ± 6.10	30.29 ± 4.30	10.20 ± 4.91	18.91 ± 8.65
Pathological grading					
Well differentiated	21	40.75 ± 7.05	29.52 ± 5.12	10.48 ± 5.58	18.55 ± 8.19
Intermediate differentiated	14	33.14 ± 6.37	25 ± 4.99	10.03 ± 4.31	19.26 ± 10.39
Poor differentiated	8	33.75 ± 8.46	26.63 ± 5.42	10.36 ± 3.79	15.85 ± 8.47

^a^Compared with ≤40 group and ≥60 group, *a* < 0.05

^b^Compared with T_3–4_ group, *b* < 0.0001

^c^Compared with T_3–4_ group, *b* < 0.0001

^d^Compared with N_1–3_ group, *b* < 0.0001

^e^Compared with N_1–3_ group, *b* < 0.0001

### Accumulation of CD3^+^CD4^+^, CD3^+^CD8^+^ T cells, CD3^−^CD19^+^ B cells, and CD3^−^CD56^+^ NK cells subsets in different OSCC development and progression

**Fig. 1:**
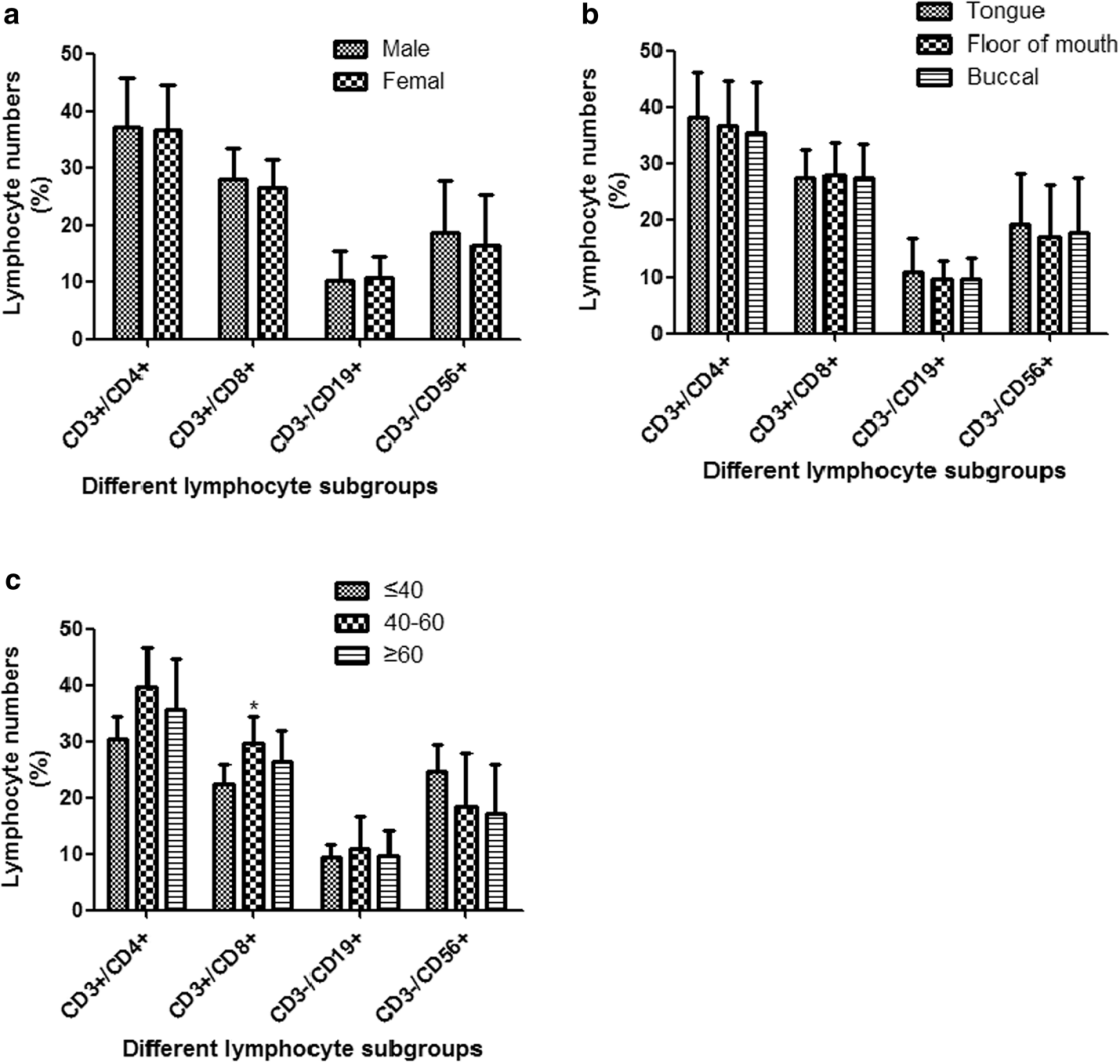
Accumulation of lymphocyte subgroups in OSCC patients with different clinical characteristics. **a** The percentage of circulating CD3^+^CD4^+^ and CD3^+^CD8^+^ T cells, CD3^−^CD19^+^ B cells, and CD3^−^CD16^+^CD56^+^ NK cells in the OSCC patients with different gender; **b** the percentage of circulating CD3^+^CD4^+^ and CD3^+^CD8^+^ T cells, CD3^−^CD19^+^ B cells, and CD3^−^CD16^+^CD56^+^ NK cells in the OSCC patients with different location of primary tumor; **c** the percentage of circulating CD3^+^CD4^+^ and CD3^+^CD8^+^ T cells, CD3^−^CD19^+^ B cells, and CD3^−^CD16^+^CD56^+^ NK cells in the OSCC patients with different age. *Error bars* indicate mean ± SD, *P < 0.05

To better understand the interactions and role of immune system in the pathogenesis of OSCC, four lymphocyte subtypes (CD3^+^CD4^+^, CD3^+^CD8^+^ T cells, CD3^−^CD19^+^ B cells, and CD3^−^CD16^+^CD56^+^ NK cells) were analyzed from peripheral blood using flow cytometer. Firstly, we analyzed the role of different clinical data on four lymphocyte subtypes in OSCC. The results demonstrated that there were no differences of lymphocyte subtypes change between female and male and among different location. There was only significant difference of CD3^+^CD8^+^ T cells numbers in 40–60 age group, compared to ≤40 age group and ≥60 age group (P < 0.05) (Fig. 1). Notably, the percentage of CD3^+^CD4^+^ T cells and CD3^+^CD8^+^ T cells distribution was significantly different in OSCC patients with different tumor size and nodal status. The percentage of CD3^+^CD4^+^ T cells distribution was significantly increased in OSCC patients with T_3–4_ tumor size (42.39 ± 5.49), compared to that with T_1–2_ tumor size (29.06 ± 3.44). The percentage of CD3^+^CD8^+^ T cells distribution was significantly increased in OSCC patients with T_3–4_ tumor size (30.69 ± 4.08), compared to that with T_1–2_ tumor size (22.65 ± 3.10). The same tendency was also observed in patients with different nodal status. The percentage of CD3^+^CD4^+^ T cells distribution was significantly increased in OSCC patients with N_1–3_ (42.29 ± 6.10), compared to that with N_0_ (30.58 ± 5.04). The percentage of CD3^+^CD8^+^ T cells distribution was also significantly increased in OSCC patients with N_1–3_ (30.29 ± 4.30), compared to that with N_0_ (24 ± 4.66).

### Dynamic distributions of CD4^+^, CD8^+^ T cells, CD19^+^ B cells, and CD56^+^ NK cells subsets in patients with different tumor size of OSCC received different treatments

**Fig. 2:**
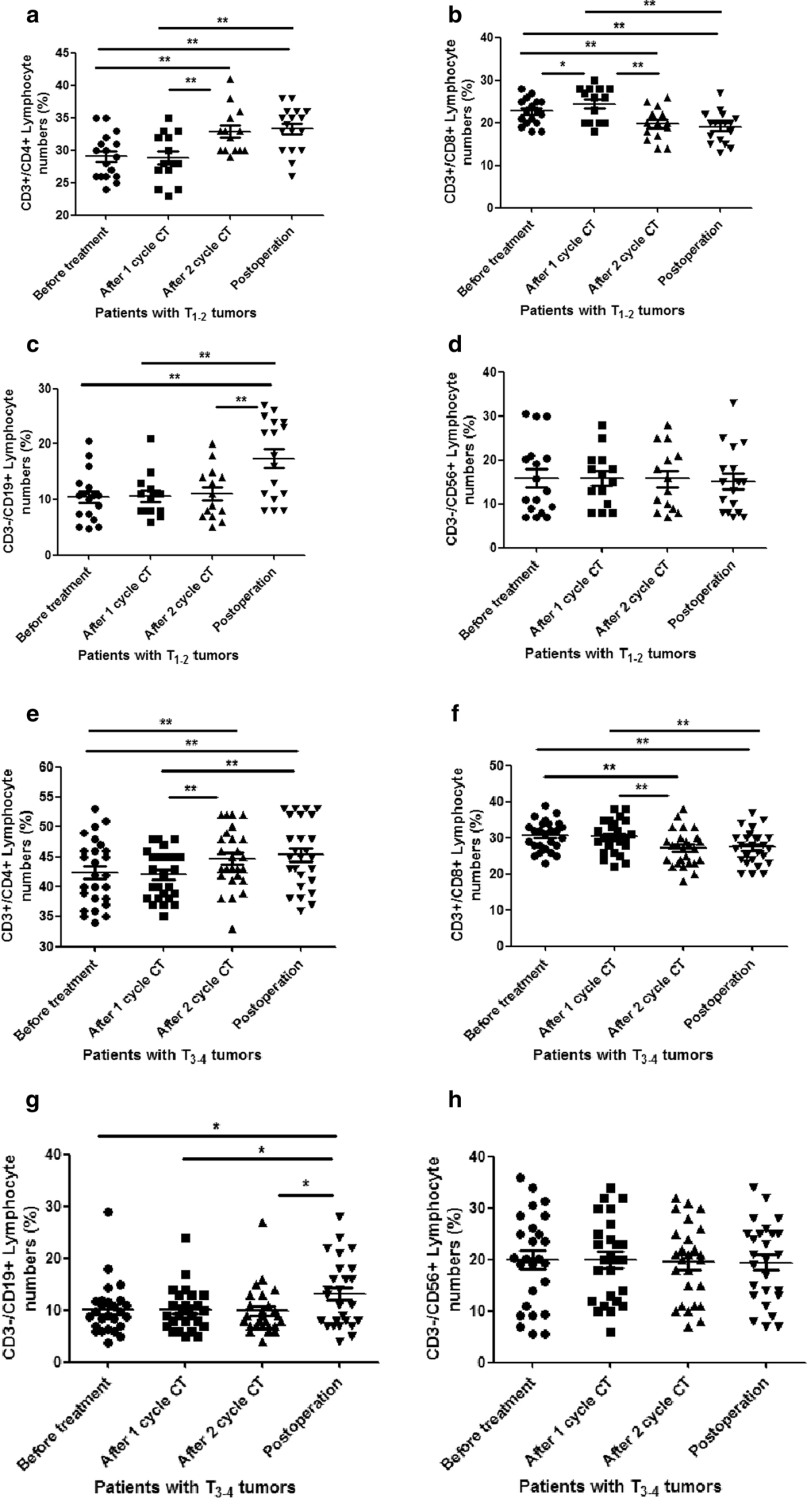
The accumulation of lymphocyte subgroups in OSCC patients with different tumor size received different treatments. **a** The accumulation of CD3^+^CD4^+^ T cells in the OSCC patients with T_1–2_ size of primary tumor received different treatments; **b** the accumulation of CD3^+^CD8^+^ T cells in the OSCC patients with T_1–2_ size of primary tumor received different treatments; **c** the accumulation of CD3^−^CD19^+^ B cells in the OSCC patients with T_1–2_ size of primary tumor received different treatments; **d** the accumulation of CD3^−^CD16^+^ CD56^+^ NK cells in the OSCC patients with T_1-2_size of primary tumor received different treatments; **e** the accumulation of CD3^+^CD4^+^ T cells in the OSCC patients with T_1–2_ size of primary tumor received different treatments; **f** the accumulation of CD3^+^CD8^+^ T cells in the OSCC patients with T_3–4_ size of primary tumor received different treatments; **g** the accumulation of CD3^−^CD19^+^ T cells in the OSCC patients with T_3–4_ size of primary tumor received different treatments; **h** the accumulation of CD3^−^CD16^+^ CD56^+^ NK cells in the OSCC patients with T_3–4_ size of primary tumor received different treatments. *Error bars* indicate mean ± SD, *P < 0.05, **P < 0.0001

To investigate the dynamic distributions of four lymphocyte subtypes in OSCC with different TNM classification received two-cycle chemotherapy and radical operation, we analyzed the percentage of CD3^+^CD4^+^, CD3^+^CD8^+^ T cells, CD3^−^CD19^+^ B cells, and CD3^−^CD16^+^CD56^+^ NK cells distribution in different time point, including 3 days before treatment, 1 week after 1 cycle chemotherapy, 1 week after 2 cycles chemotherapy, and 1 week after radical operation. According to UICC TNM classification, we analyzed the four lymphocyte subtypes distribution in patients with tumor size (T_1–2_) in four time points, compared to patients with tumor size (T_3–4_). The result demonstrated that there was no difference of CD3^+^CD4^+^ T cells distribution in patients with T_1–2_ tumors with no treatment in comparison with the 1 week after 1 cycle chemotherapy. But for patients with T_1–2_ tumors received 2 cycles CT and radical operation, the percentage of CD3^+^CD4^+^ T cells distribution was significantly increased, compared to that with no treatment and 1 week after 1 cycle CT (P < 0.0001) (Fig. 2a). Moreover, there was no difference in patients with no treatment and 1 week after 1 cycle CT, and between 1 week after 2 cycles CT and post-operation. For CD3^+^CD8^+^ T cells distribution, we observed that the T cells distribution was significantly decreased in patients with T_1–2_ received 2 cycles CT or radical operation, compared to the patients with T_1–2_ received no treatment or 1 cycle CT (P < 0.0001). Interestingly, CD3^+^CD8^+^ T cells seems to be increased in patients with T_1–2_ received 1 cycle CT, compared to that received no treatment (Fig. 2b). We also analyzed the CD3^−^CD19^+^ B cells and CD3^−^CD16^+^CD56^+^ NK cells distribution in different time points for these patients. The CD3^−^CD19^+^ B cells distribution only increased in patients with T_1–2_ received radical operation, compared to the time point of no treatment or 1–2 cycles CT (p < 0.0001) (Fig. 2c). There seems to be no difference for CD3^−^CD16^+^CD56^+^ NK cells distribution in all four time points (Fig. 2d). Almost the same tendency was observed in OSCC patients with T_3–4_ tumor size. For patients with T_3–4_ tumors received 2 cycles CT and radical operation, the percentage of CD3^+^CD4^+^ T cells distribution was significantly increased, compared to the time points of no treatment and 1 cycle CT (P < 0.0001) (Fig. 2e). Two-cycles CT and radical operation significantly decreased the percentage of CD3^+^CD8^+^ T cells, compared to the time points of no treatment or 1 cycle CT (p < 0.0001) (Fig. 2f). The radical operation is the only time point to increase the distribution of CD3^−^CD19^+^ B cells in comparison with the other three time points (p < 0.05) (Fig. 2g). The distribution of CD3^−^CD16^+^CD56^+^ NK cells was still no change in all four time points (Fig. 2h).

### Dynamic distributions of CD4^+^, CD8^+^ T cells, CD19^+^ B cells, and CD56^+^ NK cells subsets in patients with different nodal status of OSCC received different treatments

**Fig. 3:**
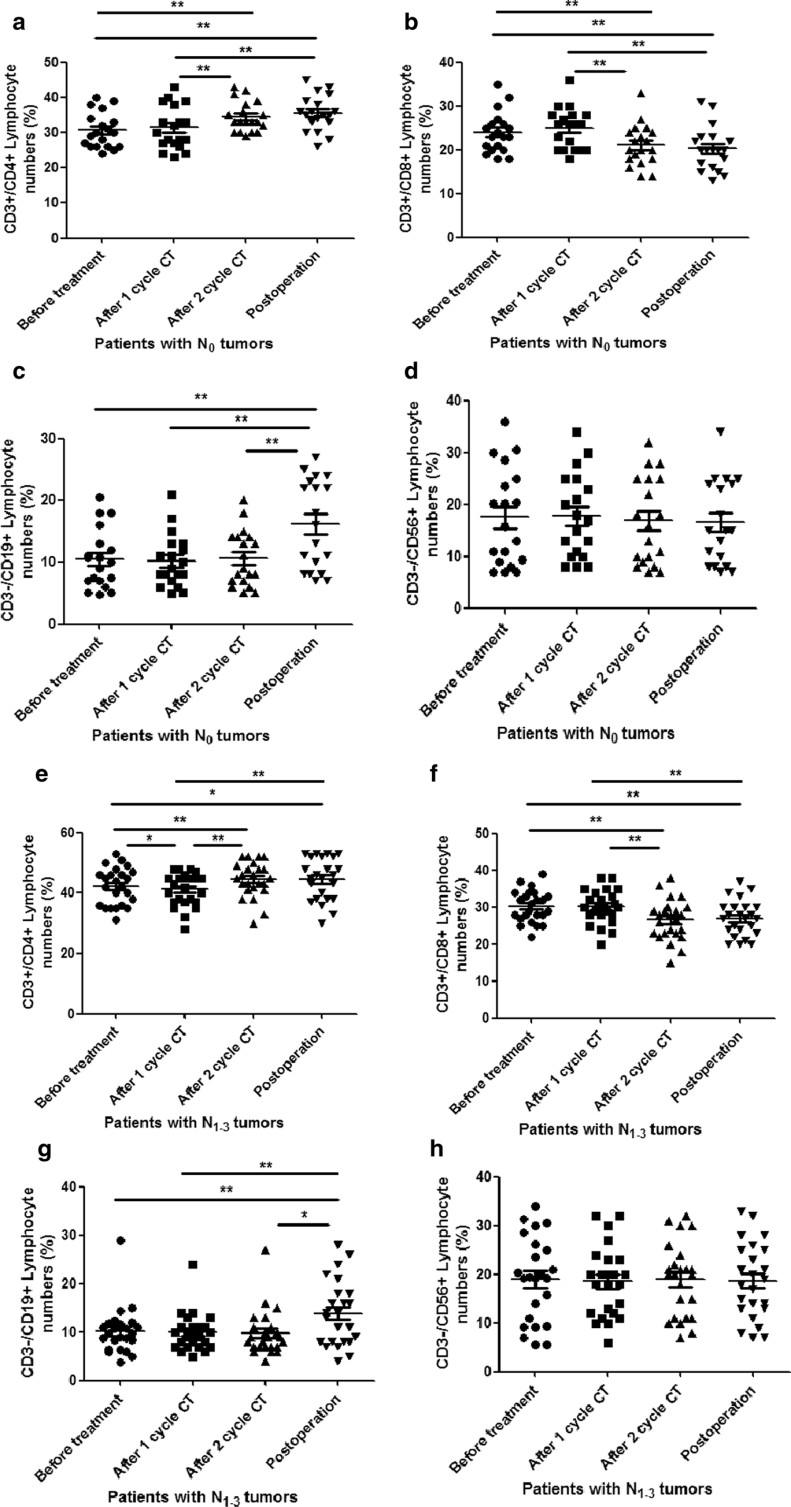
The accumulation of lymphocyte subgroups in OSCC patients with different nodal status received different treatments. **a** The accumulation of CD3^+^CD4^+^ T cells in the OSCC patients with N_0_ received different treatments; **b** the accumulation of CD3^+^CD8^+^ T cells in the OSCC patients with N_0_ received different treatments; **c** the accumulation of CD3^−^CD19^+^ B cells in the OSCC patients with N_0_ received different treatments; **d** the accumulation of CD3^−^CD16^+^ CD56^+^ NK cells in the OSCC patients with N_0_ received different treatments; **e** the accumulation of CD3^+^CD4^+^ T cells in the OSCC patients with N_1–3_ received different treatments; **f** the accumulation of CD3^+^CD8^+^ T cells in the OSCC patients with N_1–3_ received different treatments; **g** the accumulation of CD3^−^CD19^+^ T cells in the OSCC patients with N_1–3_ received different treatments; **h** the accumulation of CD3^−^CD16^+^ CD56^+^ NK cells in the OSCC patients with N_1–3_ received different treatments. *Error bars* indicate mean ± SD, *P < 0.05, **P < 0.0001

To evaluate the role of nodal status on lymphocyte subtypes distribution in OSCC patients received different treatments, we also analyzed the percentage of CD4^+^, CD8^+^ T cells, CD19^+^ B cells, and CD56^+^ NK cells distribution using flow cytometer. Similarly, the percentage of CD3^+^CD4^+^ T cells distribution was significantly increased in OSCC patients received 2 cycles CT and radical operation, compared to that received no treatment and 1 cycle CT (P < 0.0001) (Fig. 3a). There were also no differences between the patients received no treatment and 1 cycle CT, and between the patients received 2 cycles CT and radical operation. The percentage of CD3^+^CD8^+^ T cells distribution significantly decreased in OSCC patients with no lymph nodes metastasis received 2 cycles CT and radical operation, compared to that received no treatment and 1 cycle CT (Fig. 3b). The OSCC patients with no lymph nodes metastasis demonstrated that the CD3^−^CD19^+^ B cells distribution significantly increased in time point of post-operation, compared to the other three time points (p < 0.0001) (Fig. 3c). There was also no difference distribution of CD3^−^CD16^+^CD56^+^ NK cells among four time points in OSCC patients with no lymph nodes metastasis (Fig. 3d). We also analyzed the percentage of four lymphocyte subtypes distribution in OSCC patients with N_1–3_ lymph nodes metastasis received different treatments. The results demonstrated that the CD3^+^CD4^+^ T cells distribution was significantly increased in patients with N_1–3_ received 2 cycles CT and radical operation, compared to that received no treatment and 1 cycle CT (p < 0.0001) (Fig. 3e). Interestingly, after 1 cycle CT, the CD3^+^CD4^+^ T cells distribution was significantly decreased in the patients with N_2–3_, compared to that received no treatment. The percentage of CD3^+^CD8^+^ T cells distribution significantly decreased in patients with N_1–3_ received 2 cycles CT and radical operation (Fig. 3f). We also analyzed the CD3^−^CD19^+^ B cells and CD3^−^CD16^+^CD56^+^ NK cells distribution in different time points for these patients. The CD3^−^CD19^+^ B cells distribution significantly increased in patients with N_1–3_ received radical operation, compared to that received 2 cycles CT (P < 0.05), 1 cycle CT (p < 0.0001), and no treatment (p < 0.0001) (Fig. 3g). There was no difference for CD3^−^CD16^+^CD56^+^ NK cells distribution in patients with N_1-3_ in all four time points (Fig. 3h).

### Dynamic distributions of CD4^+^, CD8^+^ T cells, CD19^+^ B cells, and CD56^+^ NK cells subsets in patients with different pathologic grading of OSCC received different treatments

**Fig. 4:**
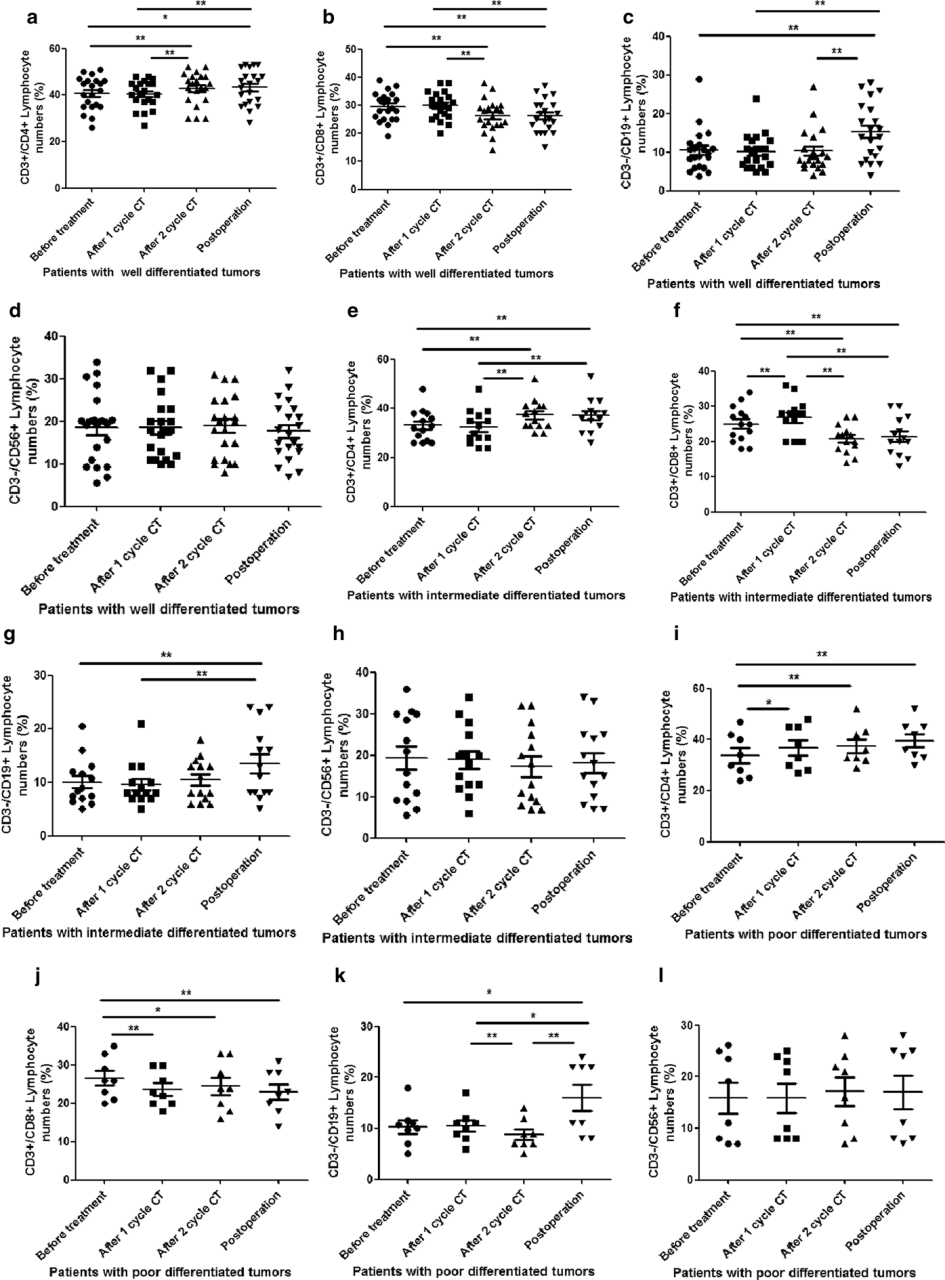
The accumulation of lymphocyte subgroups in OSCC patients with different pathological grading received different treatments. **a** the accumulation of CD3^+^CD4^+^ T cells in the OSCC patients with well differentiated tumors received different treatments; **b** the accumulation of CD3^+^CD8^+^ T cells in the OSCC patients with well differentiated tumors received different treatments; **c** the accumulation of CD3^−^CD19^+^ B cells in the OSCC patients with well differentiated tumors received different treatments; **d** the accumulation of CD3^−^CD16^+^ CD56^+^ NK cells in the OSCC patients with well differentiated tumors received different treatments; **e** the accumulation of CD3^+^CD4^+^ T cells in the OSCC patients with intermediate differentiated tumors received different treatments; **f** the accumulation of CD3^+^CD8^+^ T cells in the OSCC patients with intermediate differentiated tumors received different treatments; **g** the accumulation of CD3^−^CD19^+^ T cells in the OSCC patients with intermediate differentiated tumors received different treatments; **h** the accumulation of CD3^−^CD16^+^ CD56^+^ NK cells in the OSCC patients with intermediate differentiated tumors received different treatments. **i** the accumulation of CD3^+^CD4^+^ T cells in the OSCC patients with poor differentiated tumors received different treatments; **j** the accumulation of CD3^+^CD8^+^ T cells in the OSCC patients with poor differentiated tumors received different treatments; **k** the accumulation of CD3^−^CD19^+^ T cells in the OSCC patients with poor differentiated tumors received different treatments; **l** the accumulation of CD3^−^CD16^+^ CD56^+^ NK cells in the OSCC patients with poor differentiated tumors received different treatments. *Error bars* indicate mean ± SD, *P < 0.05, **P < 0.0001

Then we analyzed the percentage of CD4^+^, CD8^+^ T cells, CD19^+^ B cells, and CD56^+^ NK cells distribution using flow cytometer in OSCC patients with different pathological grading. For patients with well differentiated tumors, the percentage of CD3^+^CD4^+^ T cells distribution was significantly increased for patients received 2 cycles CT and radical operation, compared to the that received no treatment and 1 cycle CT (P < 0.0001, p < 0.05, respectively) (Fig. 4a). Two-cycle CT and radical operation significantly decreased the percentage of CD3^+^CD8^+^ T cells, compared to the time points of no treatment or 1 cycle CT (p < 0.0001) (Fig. 4b). The radical operation is the only time point to increase the distribution of CD3^−^CD19^+^ B cells in comparison with the other three time points (p < 0.0001) (Fig. 4c). The distribution of CD3^−^CD16^+^CD56^+^ NK cells was still no change in all four time points (Fig. 4d). The same tendency was observed in CD3^+^CD4^+^ T cells distribution in these patients received different treatments as those patients with intermediate differentiated tumors (Fig. 4e). Moreover, two-cycle CT and radical operation significantly decreased the percentage of CD3^+^CD8^+^ T cells, compared to the time points of no treatment or 1 cycle CT (p < 0.0001). However, 1 cycle CT seems to be increased the percentage of CD3^+^CD8^+^ T cells, compared to the patients received no treatment (Fig. 4f). There was significant difference of CD3^−^CD19^+^ B cells between patients with intermediate differentiated tumors received radical operation and that received no treatment, and between patients with intermediate differentiated tumors received radical operation and that received 1 cycle CT (Fig. 4g). The distribution of CD3^−^CD16^+^CD56^+^ NK cells was still no change in all four time points (Fig. 4h). At last, we analyzed that the CD4^+^, CD8^+^ T cells, CD19^+^ B cells, and CD56^+^ NK cells distribution using flow cytometer in OSCC patients with poor differentiated tumors received different treatments. The percentage of CD3^+^CD4^+^ T cells distribution was significantly increased in patients received 1cycle CT, 2 cycles CT and radical operation, compared to the time points of no treatment (P < 0.0001) (Fig. 4i). The percentage of CD3^+^CD8^+^ T cells distribution was also significantly decreased in patients with poor differentiated tumors received 1 cycle CT (p < 0.0001), 2 cycles CT (p < 0.05), and radical operation (p < 0.0001), compared to that patients received no treatment. Moreover, the radical operation significantly increased the distribution of CD3^−^CD19^+^ B cells in patients with poor differentiated tumors, compared to that received no treatment and 1 cycle CT (Fig. 4j). However, 2 cycles CT seems to be decreased the distribution of CD3^−^CD19^+^ B cells in patients with poor differentiated tumors, compared to that received 1 cycle CT and radical operation (Fig. 4k). The distribution of CD3^−^CD16^+^CD56^+^ NK cells was still no change in all four time points (Fig. 4l).

### Association of CD4^+^/CD8^+^ ratio with OSCC patients received different treatments

**Fig. 5:**
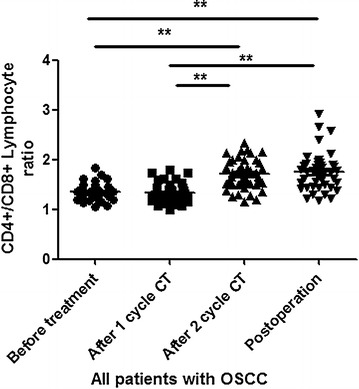
The CD4^+^/CD8^+^ ratio in OSCC patients received different treatments. *Error bars* indicate mean ± SD, **P < 0.0001

To evaluate the effect of different treatments on patients with OSCC, we also analyze the CD4^+^/CD8^+^ ratio in all OSCC patients received no treatment, 1 cycle CT, 2 cycles CT, and radical operation. The 2 cycles CT and radical operation indeed increased the CD4^+^/CD8^+^ ratio in OSCC patients, compared to that received no treatment or 1 cycle CT (P < 0.0001). There was no significant difference between patients received no treatment and patients received 1 cycle CT, between patients received 2 cycles CT and patients received radical operation (Fig. 5). These data suggested that the 2 cycles CT and radical operation treatment may be essential to increase the effect of treatment for the patients with OSCC.

## Discussion

Kuhn et al. reported that abnormal T lymphocyte subgroups were found in peripheral blood, marked by decreased CD3^+^CD4^+^ expression and increased CD3^+^CD8^+^ expression (Kuhn et al. 2013). However, comprehensive understanding of the role of CD4^+^ T cells in anti-tumor immunity is challenging in tumor immunology research and the results have been controversial. Besides the traditional functions of Th1 and Th2 cells in helping tumor-specific CD8^+^ T cells and B cells, recent discovery of Th17 and Treg cells has not only resulted in an explosion of cancer immunological research but also markedly changed our conventional thinking of the role of CD4^+^ T cells in the pathologenesis of cancer development. In this study, OSCC patients in advanced group (T_3–4_/N_1–3_) was significantly higher in expression of CD3^+^CD4^+^ cells in peripheral blood than early group (T_1–2_/N_0_). It’s reported that in early tumor stages, Th1 cells are the dominant population of CD4^+^ T cells, perhaps important for immunosurveillance; while in the advanced tumor stages, FoxP3+ Treg and Th17 cells become the dominant populations. It is well recognized that tumor-infiltrating Treg cells are a major obstacle for the success of tumor immunity and immunotherapy (Curiel 2008; Perez et al. 2007; Peng et al. 2007). Huang et al. also reported that the intra-tumoral CD4^+^ T cells numbers were positively correlated with advanced tumor stage, large tumor size, and positive tumor metastasis, but were inversely correlated with survival of breast cancer patients (Huang et al. 2015). Therefore, this study maybe suggested that in the late tumor stage, CD4^+^ T cells become more important for promoting tumor growth. The expression of CD3^+^CD8^+^ seemed to be decreased in early group (T_1–2_/N_0_), compared to that in advanced group (T_3–4_/N_1–3_). However, except for group between ≤40 age group and ≥60 age group in comparison with 40–60 age group, there were no significant differences in the expression of CD3^+^CD4^+^ and CD3^+^CD8^+^ in OSCC patients with different pathological patterns, genders, and locations.

Accumulating research findings suggested that there are multiple antitumor T cell clones in the peripheral blood of patients with lymphoma, but these T lymphocytes fail to retain effective antitumor properties (Yin et al. 2010, 2011). Interestingly, Yin et al. showed that imbalance T-lymphocyte subsets occurs in patients with lymphoma during different chemotherapy stages, and these T-lymphocyte subsets gradually return to normal by 3 months after chemotherapy (Yin et al. 2014). Given the poor prognosis of OSCC, the patients with OSCC (T_2–4_/N_1–3_) received 2 cycles TP regime chemotherapy and radical operation, including extensive resection of the primary tumor, partial resection of maxillary/mandible, and functional neck dissection (Level I–V). To evaluate the dynamic effect of chemotherapy or operation on patients with OSCC, the lymphocytes subgroups were analyzed using flow cytometer. Interesting, 2 cycles TP regime chemotherapy and radical operation indeed significantly increased the expression of CD3^+^CD4^+^ T cells and decreased the expression of CD3^+^CD8^+^ T cells in almost all OSCC patients. The results suggested that the 2 cycles CT and radical operation were essential to improve the prognosis of OSCC patients. It is suggested that CD4^+^/CD8^+^ ratio is crucial to be kept in a dynamic balance, in terms of maintaining the immunological function stable (Dou et al. 2013). The ratio decreased is reported to link with a low immunological function (Erdem et al. 2012; Nugroho et al. 2012). In this study, The patients with OSCC received 2 cycles CT or radical operation had a significantly higher level of CD4^+^/CD8^+^ ratio compared with that received no treatment or 1 cycle CT. NK cell is also designated as natural killer cells. NK cell could directly destroy tumor cells, and reduced level of NK cell will lead to decline of immunological function (Wang et al. 2013). Our results showed that there is no difference of expression of CD3^–^CD16^+^CD56^+^ NK cell in OSCC patients received different treatments, suggesting no significant influence of different treatments of NK cells on patients with OSCC. The CD19 molecule is a phenotypic marker of B cells. In EBV-associated disease, B cells are the principal targets of viral infection and play a significant role in the anti-tumor humoral responses by differentiating into antibody producing plasma B cells. The prognostic value of CD19^+^ B cells also has been observed in breast cancer and acute myeloid leukemia (Tredan et al. 2013; Iriyama et al. 2013). The high percentage of circulating CD19^+^ B cells alone correlated positively with better PFS of nasopharyngeal carcinoma (Xu et al. 2014). In our study, the expression of CD19^+^ B cells were only increased in OSCC patients received radical operation with different T stage, nodal status and pathological grading. There is no relationship between 2 cycles TP regime chemotherapy and the expression of CD19^+^ B cells. The mechanism need to be further explored.

## Conclusion

In this study, the expression of CD4^+^ T cells were significantly increased in advanced tumor stage, large tumor size and positive lymph nodes metastasis, compared to that in early group. 2 cycles TP regime chemotherapy and radical therapy may be essential to increase the effects of anti-tumor immunity on patients with OSCC. However, the positive correlation between circulating CD19^+^ B cells and radical operation in patients with OSCC still need to be further explored in future study. The analysis of lymphocyte subgroups in peripheral blood detected by flow cytometry suggested that the lymphocyte subgroups may be the prognostic factor of OSCC.
